# Tonsillectomy increases the risk of retropharyngeal and parapharyngeal abscesses in adults, but not in children: A national cohort study

**DOI:** 10.1371/journal.pone.0193913

**Published:** 2018-03-06

**Authors:** So Young Kim, Chanyang Min, Woo Hyun Lee, Hyo Geun Choi

**Affiliations:** 1 Department of Otorhinolaryngology-Head & Neck Surgery, CHA Bundang Medical Center, CHA University, Seongnam, Korea; 2 Hallym Data Science Laboratory, Hallym University College of Medicine, Anyang, Korea; 3 Department of Otorhinolaryngology, National Police Hospital, Seoul, Korea; 4 Department of Otorhinolaryngology-Head & Neck Surgery, Hallym University College of Medicine, Anyang, Korea; Nantes University Hospital, FRANCE

## Abstract

**Objectives:**

The purpose of this study is to evaluate the risk of retropharyngeal and parapharyngeal abscesses (deep neck infection) after tonsillectomy in Koreans using national cohort data.

**Methods:**

Using the national cohort study from the Korean Health Insurance Review and Assessment Service, participants who had undergone a tonsillectomy (5,299) and control participants (21,196) were selected and matched 1:4 (for age, sex, income, region of residence, and pre-operative upper respiratory infection visits). The Cox-proportional hazard model was used. A crude model and an adjusted model for age, sex, income, region of residence, hypertension, diabetes, and dyslipidemia were used in this analysis. For the subgroup analyses, the participants were divided into 2 groups: children (≤ 14 years old) and adolescents and adults (≥ 15 years old).

**Results:**

The adjusted hazard ratio of deep neck infection after tonsillectomy was 1.43 (95% confidence interval, CI = 1.18–1.72, P < 0.001). In subgroup analysis, this ratio was 1.12 (95% CI = 0.86–1.47, P = 0.390) in children and 1.87 (95% CI = 1.43–2.45, P < 0.001) in adolescents and adults. The crude hazard ratios were almost the same as the adjusted ratios.

**Conclusion:**

The risk of deep neck infection was higher in the tonsillectomy group. The subgroup analysis showed a similar finding in the adolescent and adult group but not in the child group.

## Introduction

Tonsillectomy with/without adenoidectomy is one of the most common surgeries in children worldwide [1]. The rate of tonsillectomy in those under 18 years old is 3.4 to 4.8 per 1,000 people in the US [2, 3]. Our previous study reported this rate to be 2.58 per 1,000 people under 16 years old in Korea [4]. Tonsillectomy is commonly indicated for chronic tonsillar hypertrophy, chronic tonsillitis, or obstructive sleep apnea [1, 4].

The tonsils and adenoids are lymphoid tissues of Waldeyer’s ring. They are involved in the defense against antigens of the upper respiratory tract [5, 6]. The uptake of antigens by M-cells in the crypt epithelium results in the generation and dissemination of antigen-specific memory and mainly dimeric IgA-producing effector B-lymphocytes [6]. Therefore, elimination of tonsil tissue could alter the immune response. Retropharyngeal or parapharyngeal infection (deep neck infection) is a serious condition involving the parapharyngeal space of the neck, which is enclosed by deep fascia, and originates from an upper aerodigestive tract infection [7]. However, few studies have reported the possibility of deep neck infection after tonsillectomy.

We searched Pubmed and Embase using the terms ‘tonsillectomy, deep neck infection, retropharyngeal abscess, and/or parapharyngeal abscess’ and limited our search to articles written in English. We found only two studies that reported an association between tonsillectomy and deep neck infection. One case-control study reported that the odds ratio (OR) of a deep neck infection after tonsillectomy was 7.10 (95% confidence interval, CI = 2.52–19.93) [8]. The other cohort study reported that patients with a previous tonsillectomy have a 1.71 times greater risk of deep neck infection (95% CI = 1.13–2.59) [9]. However, in the previous studies, the “participants with tonsillectomy” group and the “participants without tonsillectomy” group were not well matched for their susceptibility to upper respiratory infection (URI). Because the patients who have undergone tonsillectomy and are vulnerable to URI might also have a higher risk of deep neck infection, the difference in susceptibility to a URI could be decisive.

The purpose of this study is to evaluate the risk of deep neck infection after tonsillectomy in Korean patients using the national cohort data. We matched the tonsillectomy participants to those without tonsillectomy (controls) for age, sex, income, region of residence, and previous history of URI for 2 years before the tonsillectomy. This study could provide useful information that has not been reported by previous studies.

## Materials and methods

### Study population and data collection

The ethics committee of Hallym University (2014-I148) approved the use of these data. Written informed consent was exempted by the Institutional Review Board.

This national cohort study relies on data from the Korean Health Insurance Review and Assessment Service—National Patient Sample (HIRA-NPS). The Korean National Health Insurance Service (NHIS) selects samples directly from the entire population database to prevent non-sampling errors. Approximately 2% of the samples (one million) were selected from the entire Korean population (50 million). These selected data can be classified at 1,476 levels (age [18 categories], sex [2 categories], and income level [41 categories]) using randomized stratified systematic sampling methods via proportional allocation to represent the entire population. After data selection, the appropriateness of the sample was verified by a statistician who compared the data from the entire Korean population to the sample data. The details of the methods used to perform these procedures are provided by the National Health Insurance Sharing Service [10]. This cohort database included (i) personal information, (ii) health insurance claim codes (procedures and prescriptions), (iii) diagnostic codes using the International Classification of Disease-10 (ICD-10), (iv) socio-economic data (residence and income), and (v) medical examination data for each participant over a period ranging from 2002 to 2013.

Because all Korean citizens are recognized by a 13-digit resident registration number from birth to death, exact population statistics can be determined using this database. It is mandatory for all Koreans to enroll in the NHIS. All Korean hospitals and clinics use the 13-digit resident registration number to register individual patients in the medical insurance system. Therefore, the risk of overlapping medical records is minimal, even if a patient moves from one place to another. Moreover, all medical treatments in Korea can be tracked without exception using the HIRA system.

### Selection of participants

Of 1,025,340 cases with 114,369,638 medical claim codes, we included participants who underwent a tonsillectomy (claim code: Q2300) from 2004 to 2011 (n = 5,695). Among these, participants who underwent a tonsillectomy for malignancy were excluded (n = 44).

**Fig 1:**
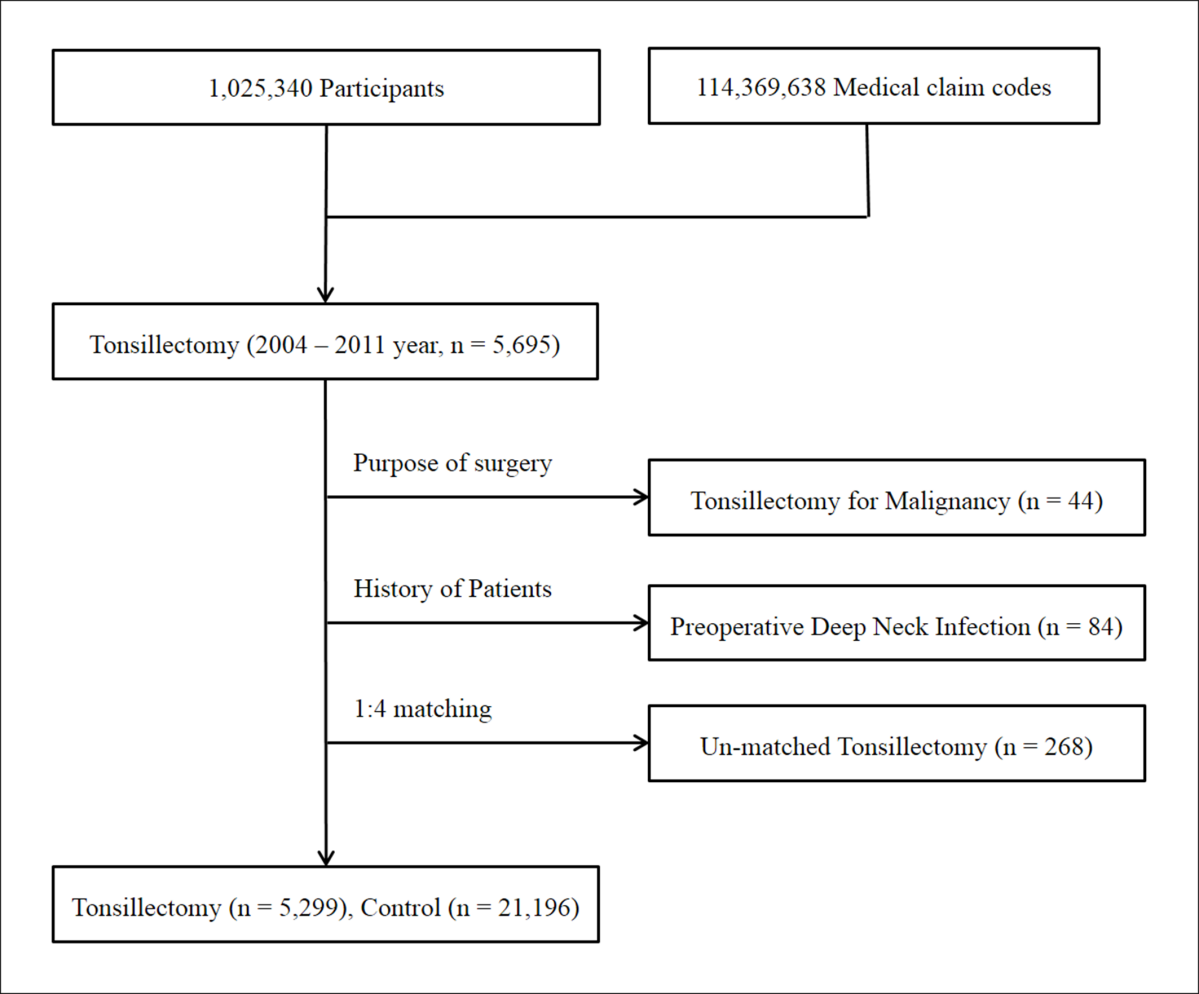
A schematic illustration of the participant selection process used in the present study. Of a total of 1,025,340 participants, 5,695 tonsillectomy participants were selected. Individuals who underwent a tonsillectomy for malignancy were excluded (n = 44). Tonsillectomy participants with a history of pre-operative deep neck infection were excluded (n = 84). The tonsillectomy participants were matched 1:4 with a control group that had not undergone a tonsillectomy. Unmatched tonsillectomy participants were excluded (n = 268). Ultimately, 5,299 tonsillectomy participants and 21,196 control participants were included.

We defined retropharyngeal or parapharyngeal abscess (deep neck infection) using the following ICD-10 codes: J39.0 (retropharyngeal and parapharyngeal abscess) and J39.1 (other abscess of the pharynx). Participants with a history of retropharyngeal or parapharyngeal abscess (deep neck infection) before the tonsillectomy were excluded (n = 84). Hence, only participants who underwent tonsillectomy for benign causes (e.g., chronic tonsillitis, chronic tonsillar hypertrophy, and obstructive sleep apnea) were included. Participants who underwent a tonsillectomy were matched 1:4 with participants (control group) who had not undergone a previous tonsillectomy from 2002 to 2013 among this cohort. The control participants were extracted from 1,025,340 participants. The matches were processed for age, group, sex, income group, region of residence, and 2-year pre-operative history of URI. To prevent selection bias when selecting the matched participants, the control group participants were sorted using a random order and were then selected based on this order. The matched control participants were assumed to have been examined at the same time (index date = tonsillectomy date) as each matched tonsillectomy participant. In the control group, participants with a history of deep neck infection before the index date were excluded. We hypothesized that tonsillectomy participants and control participants were involved at the index date and then they were followed up to December 2013. Tonsillectomy participants for whom we could not identify four matching participants were excluded (n = 268). Ultimately, 1:4 matching resulted in the inclusion of 5,299 tonsillectomy participants and 21,196 control participants (Fig 1). However, the participants were not matched based on their past medical histories (hypertension, diabetes, and dyslipidemia).

### Variables

The age groups were classified into 5-year intervals: 0–4, 5–9, 10–15…, and 80+ years old. A total of 17 age groups were designated. The income groups were initially divided into 41 classes (one health aid class, 20 self-employment health insurance classes, and 20 employment health insurance classes). These groups were re-categorized into 11 classes (class 1 [lowest income]-11 [highest income]). The region of residence was divided into 16 areas according to administrative district. These regions were regrouped into urban (Seoul, Busan, Daegu, Incheon, Gwangju, Daejeon, and Ulsan) and rural (Gyeonggi, Gangwon, Chungcheongbuk, Chungcheongnam, Jeollabuk, Jeollanam, Gyeongsangbuk, Gyeongsangnam, and Jeju) areas.

We defined URIs using the following ICD-10 codes: J00 (acute nasopharyngitis) and J02 (acute pharyngitis) through J069 (acute upper respiratory infection). The number of visits to clinics or hospitals for a URI was counted for each year. Pre-operative URI visits were counted for 2 years. The participants were followed up for 2 to 10 years (mean 76.7 ± 27.3 months).

The past medical histories of participants were evaluated using ICD-10 codes. To obtain an accurate diagnosis, participants were considered to have hypertension (I10 and I15), diabetes (E10-E49), or dyslipidemia (E78) if they were treated ≥ 2 times.

### Statistical analyses

A chi-squared test was used to compare the age, sex, income, region of residence, and rates of hypertension, diabetes, dyslipidemia, and deep neck infection between the tonsillectomy and control groups. A Kaplan-Meier curve was used to analyze the cumulative probability of deep neck infection after tonsillectomy. The Cox-proportional hazard model was used to analyze the hazard ratio of deep neck infection after tonsillectomy. A crude model and an adjusted model for age, sex, income, region of residence, hypertension, diabetes, and dyslipidemia were used in this analysis. The 95% CI was calculated. For the subgroup analyses, the participants were divided into 2 groups: children (≤ 14 years old) and adolescents and adults (≥ 15 years old). Two-tailed analyses were conducted, and P values less than 0.05 were considered to indicate statistical significance. The results were statistically analyzed using SPSS v. 21.0 (IBM, Armonk, NY, USA).

## Results

Because the tonsillectomy group was matched 1:4 for age, sex, income, residence, and previous history of URI, the age, sex, income, and residence of participants were the same in the tonsillectomy and control groups (P = 1.000, Table 1). The number of previous URIs was identical in both the tonsillectomy and control groups (mean = 5.56 times, standard deviation = 5.31 times). However, the rates of hypertension, diabetes, and dyslipidemia were higher in the tonsillectomy group (P < 0.001). The rate of deep neck infection was not different between the children and the adolescents and adults (P = 0.115, S1 Table). The mean time between tonsillectomy and infection was 53.0 ± 30.0 months.

**Table 1:** General characteristics of participants.

Characteristics		The Number of participants		
		Tonsillectomy group	Control group	P-value
Age (years old) (n, %)				1.000
	0–4	1,548 (29.2)	6,192 (29.2)	
	5–9	1,007 (19.0)	4,028 (19.0)	
	10–14	618 (11.7)	2,472 (11.7)	
	15–19	459 (8.7)	1,836 (8.7)	
	20–24	420 (7.9)	1,680 (7.9)	
	25–29	365 (6.9)	1,460 (6.9)	
	30–34	297 (5.6)	1,188 (5.6)	
	35–39	199 (3.8)	796 (3.8)	
	40–44	159 (3.0)	636 (3.0)	
	45–49	112 (2.1)	448 (2.1)	
	50–54	48 (0.9)	192 (0.9)	
	55–59	38 (0.7)	152 (0.7)	
	60–64	24 (0.5)	96 (0.5)	
	65–69	4 (0.1)	16 (0.1)	
	70–74	0 (0.0)	0 (0.0)	
	75–79	1 (0.0)	4 (0.0)	
Sex (n, %)				1.000
	Male	3,124 (59.0)	1,2496 (59.0)	
	Female	2,175 (41.0)	8,700 (41.0)	
Income (n, %)				1.000
	1 (lowest)	36 (0.7)	144 (0.7)	
	2	196 (3.7)	784 (3.7)	
	3	235 (4.4)	940 (4.4)	
	4	336 (6.3)	1,344 (6.3)	
	5	449 (8.5)	1,796 (8.5)	
	6	507 (9.6)	2,028 (9.6)	
	7	605 (11.4)	2,420 (11.4)	
	8	717 (13.5)	2,868 (13.5)	
	9	773 (14.6)	3,092 (14.6)	
	10	765 (14.4)	3,060 (14.4)	
	11 (highest)	680 (12.8)	2,720 (12.8)	
Region of residence (n, %)				1.000
	Urban	2,400 (45.3)	9,600 (45.3)	
	Rural	2,899 (54.7)	11,596 (54.7)	
Hypertension (n, %)				< 0.001*
	Yes	374 (7.1)	1,085 (5.1)	
	No	4,925 (92.9)	20,111 (94.9)	
Diabetes (n, %)				< 0.001*
	Yes	209 (3.9)	626 (3.0)	
	No	5,090 (96.1)	20,570 (97.0)	
Dyslipidemia (n, %)				< 0.001*
	Yes	419 (7.9)	1,154 (5.4)	
	No	4,880 (92.1)	20,042 (94.6)	
Deep neck infection (n, %)				< 0.001*
	Yes	148 (2.8)	415 (2.0)	
	No	5,151 (97.2)	20,781 (98.0)	

* Chi-square test, Significance at P < 0.05

**Fig 2:**
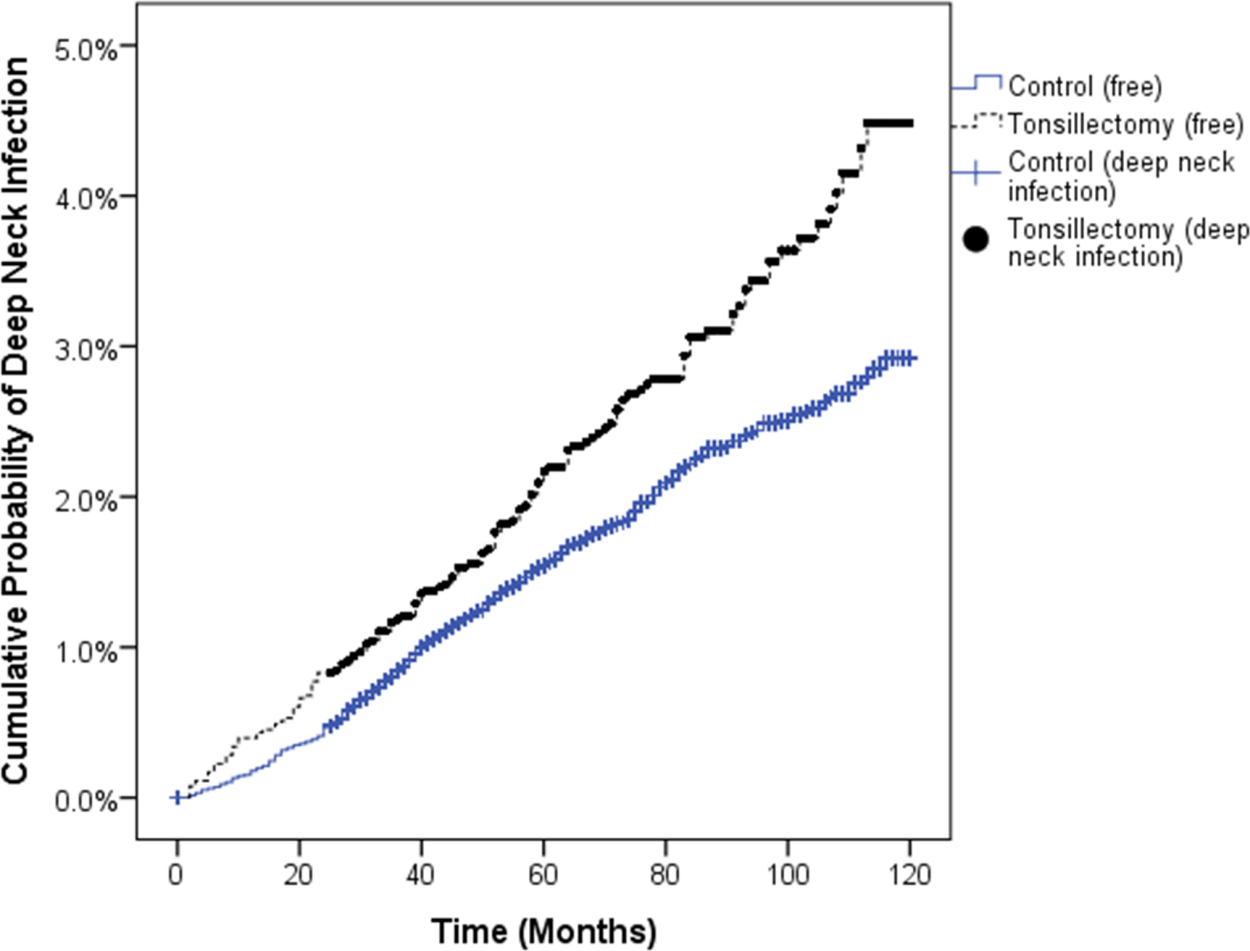
The cumulative probability of deep neck infection in the tonsillectomy group compared to the control group.

The adjusted hazard ratio of deep neck infection after tonsillectomy was 1.43 (95% CI = 1.18–1.72, P < 0.001). In the subgroup analysis, the ratio was 1.12 (95% CI = 0.86–1.47, P = 0.390) in children and 1.87 (95% CI = 1.43–2.45, P < 0.001) in adolescents and adults. The crude hazard ratios were nearly equal to the adjusted ratios (Table 2). The cumulative probability of deep neck infection was significantly higher in the tonsillectomy group than in the control group (log rank test: P < 0.001, Fig 2).

**Table 2:** Crude and adjusted hazard ratios (95% confidence interval) of tonsillectomy for deep neck infection.

Characteristics		Deep neck infection			
		Crude	P-value	Adjusted†	P-value
Total			< 0.001*		< 0.001*
	Tonsillectomy	1.43 (1.19–1.73)		1.43 (1.18–1.72)	
	Control	1.00		1.00	
Children			0.384		0.390
	Tonsillectomy	1.13 (0.86–1.47)		1.12 (0.86–1.47)	
	Control	1.00		1.00	
Adolescents & Adults			< 0.001*		< 0.001*
	Tonsillectomy	1.90 (1.45–2.48)		1.87 (1.43–2.45)	
	Control	1.00		1.00	

* Cox-proportional hazard regression model, Significance at P < 0.05

† Adjusted model for age, sex, income, region of residence, hypertension, diabetes, and dyslipidemia

## Discussion

The risk of deep neck infection was 1.43 times higher in the tonsillectomy group than in the control group. The risk was 1.87 times higher in adolescents and adults. However, the risk of deep neck infection was not statistically significant in children.

Our study addresses the limitations of previous studies; prior studies did not match participants for pre-operative history of URI [8, 9]. Thus, an increased risk of deep neck infection is possible due to the difference in susceptibility to URI in the tonsillectomy group. However, we found a consistent result after matching for previous history of URI. Therefore, the difference in susceptibility to URI between the tonsillectomy and control groups could not explain the increased risk of deep neck infection after tonsillectomy.

The alteration of immune function after tonsillectomy could be an explanation for the increased risk of deep neck infection. However, previous reports on the relationship between tonsillectomy and immune function have been controversial. We found two studies: one study reported that tonsillectomy does not compromise the immune function of children during short-term (3 months) and long-term (54 months) follow-up [11]. Another randomized controlled study stated that immunoglobulin levels were not different, except for IgA, in children [12]. However, another patient-controlled study described a decrease in the level of secretory IgA more than 20 years after tonsillectomy [13]. Therefore, evidence in support of this hypothesis (that a decrease in immune function after tonsillectomy may increase the risk of deep neck infection) is still lacking.

If risk of URI can increase after tonsillectomy, this could explain the increase in deep neck infection, in that frequent URI is a risk factor for deep neck infection [14]. However, a recent Cochrane review concluded that sufficient evidence does not exist to indicate that a tonsillectomy changes the number sore throat episodes [15]. Our recent study also reported that tonsillectomy does not alter the frequency of URI [16]. Therefore, the change in URI frequency after tonsillectomy cannot explain the increase in deep neck infections.

We suggest that the difference in the rate of deep neck infection between the children and the adolescents and adults could be responsible for the difference in deep neck infection observed after tonsillectomy between these two groups. However, the rates of deep neck infection were not significantly different between the children’s group and the adolescent and adult group (S1 Table).

We found that the risk of deep neck infection was increased only in the adolescent and adult group. The decreased IgA level in tonsillectomy patients could be compensated for if the patient experienced frequent URIs [12] because the remaining mucosa-associated lymphoid tissue could compensate for the loss of the tonsils [12]. We suggest that the difference in compensation ability between children and adults could affect the increased risk of deep neck infection in adolescents and adults. Children who are susceptible to URI might compensate for immunological function, while adolescents and adults might not have this compensatory ability. In a previous study [9], the risk of deep neck infection was higher in tonsillectomy patients with sleep apnea or hypertrophy of the tonsils [9], but the authors did not provide an explanation for this finding. We suggest that the adult group might undergo tonsillectomy for sleep apnea more often than for chronic tonsillitis, as seen in a previous study [15]; thus, they might have a lower likelihood of compensating for the decreased immunological function, which could be an explanation for the increased risk of deep neck infection in the adolescent and adult group.

One advantage of this study is the large number of study participants (n = 26,495). We followed up the tonsillectomy group for 2 to 9 years. As stated previously, we determined and matched for previous URI history. This information was obtained from the medical records of the HIRA. Therefore, there is no possibility of recall bias in the present study. The HIRA data include all citizens of the nation, without exception. Therefore, no participants are missing during any of the follow-up periods. We matched the controls according to age, sex, income, and region of residence. Income and region matching were important because these are determinants of medical procedures. Income levels can be determined very accurately using the Korean NHIS because a patient’s premium is based on their income. Our study results are therefore representative of the entire Korean population because the data were selected from a database that covers the entire population, and the representativeness of the data was verified by a statistician.

Our study has several limitations. We included participants who underwent tonsillectomy for chronic tonsillar hypertrophy or OSA because we could not determine the purpose of tonsillectomy in each participant. We did not have sufficient explanations for our results. Because we followed up participants from 2002 to 2013, participants who underwent tonsillectomy before the year 2002 might have been included in the control group. However, considering the annual incidence of tonsillectomy in Korea, the possibility is very low. If tonsillectomy histories were recorded for more than 20 years, the incidence might be less than 5%. We did not match the participants according to the presence of hypertension, diabetes, and dyslipidemia, and the rates of these diseases were higher in the tonsillectomy group. However, the results of the crude and adjusted models were almost equal. Because most of the participants (68.6%) were younger than 20 years old and the prevalence of these diseases was very low in this study, these factors did not affect the conclusion. Incidence of retropharyngeal and parapharyngeal abscess was much higher than the reported [17]. The difference of inclusion criteria and diagnostic criteria might affected it.

## Conclusion

The adjusted hazard ratio of deep neck infection was 1.43 times higher in the tonsillectomy group than in the control group. In subgroup analysis of adolescents and adults, this ratio was 1.87 times higher in the tonsillectomy group. However, the risk of deep neck infection after tonsillectomy did not increase in children.

## Supporting information

S1 Table The rate of deep neck infection in the children’s group and the adolescent and adult group. (DOCX)
