# Supplementary material for: Tonsillectomy increases the risk of retropharyngeal and parapharyngeal abscesses in adults, but not in children: A national cohort study
Source: PLoS One. 2018 Mar 6;13(3):e0193913. doi: 10.1371/journal.pone.0193913 (PMC5839582; doi:10.1371/journal.pone.0193913)
Supplement: S1 Table — (DOCX) [file pone.0193913.s001.docx]

**S1 Table** The rate of deep neck infection between children and adolescent and adults

|  | Deep neck infection (-) | Deep neck infection (+) | P-value |
| --- | --- | --- | --- |
| Children | 15,546 (98.0%) | 319 (2.0%) | 0.115 |
| Adolescents & Adults | 10,386 (97.7%) | 244 (2.3%) |  |

* Chi-square test, Significance at P < 0.05
